# Indocyanine green potentiated paclitaxel nanoprodrugs for imaging and chemotherapy

**DOI:** 10.1002/EXP.20220008

**Published:** 2022-06-04

**Authors:** Xiujuan Xiang, Xuan Feng, Shaojin Lu, Bowen Jiang, Dengyuan Hao, Qing Pei, Zhigang Xie, Xiabin Jing

**Affiliations:** ^1^ State Key Laboratory of Polymer Physics and Chemistry, Changchun Institute of Applied Chemistry Chinese Academy of Sciences Changchun Jilin China; ^2^ University of Science and Technology of China Hefei China

**Keywords:** indocyanine green, prodrug, self‐assembly

## Abstract

Self‐assembled prodrug nanoparticles with tumor‐responsive capacity have great potential in tumor visualization and treatment. However, the nanoparticle formulas usually contain multiple components, especially polymeric materials, which result in various potential issues. Herein, we report an indocyanine green (ICG)‐driven assembly of paclitaxel prodrugs integrating near‐infrared fluorescence imaging and tumor‐specific chemotherapy. By feat of the hydrophilic merit of ICG, paclitaxel dimer could form more uniformly monodispersed nanoparticles. This two‐in‐one strategy reinforces the complementary advantages, resulting in superior assembly behavior, robust colloidal stability, enhanced tumor accumulation as well as desirable near‐infrared imaging and in vivo feedback of chemotherapy. The in vivo experiments validated the prodrug activation at tumor sites as evidenced by enhanced fluorescence intensity, potent tumor growth suppression, and reduced systemic toxicity compared with commercial Taxol. The universality of ICG potentiated strategy toward photosensitizers and fluorescence dyes was confirmed. This presentation provides deep insight into the feasibility of constructing clinical‐close alternatives for improving antitumor efficacy.

## Graphical abstract

Clinically approved indocyanine green (ICG) is utilized to develop simple prodrug nanoformulations. The ICG‐driven assembly strategy endows paclitaxel prodrugs improved assembled behavior and enhanced colloidal stability, and the developed nanoparticles possess tumor‐specific imaging and chemotherapy with in vivo therapeutic feedback.

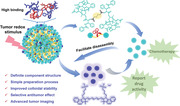

## 1 INTRODUCTION

Most anticancer chemotherapeutics are hydrophobic and exhibit unsatisfied pharmacokinetic performance.^[^ ^1^ ^]^ Harnessing the advantage of nanotechnology to formulate drugs into various vectors is an effective approach to overcome this dilemma.^[^ ^2^ ^]^ However, low drug loading and resistance of protein adsorption require addition of complicated carrier materials, which not only makes preparation process complicated, but also provokes superfluous concerns on potential carriers‐relevant toxicity and immunogenic reaction.^[^ ^3^ ^]^ For example, the drug content of clinical representative Abraxane (albumin‐bound paclitaxel (PTX) formulation) is 10 wt%, and their preparation highly depends on the high‐pressure homogenizer. Simplification of the major components is the most direct and practicable strategy to optimize nanoformulations. Drug self‐assembly has emerged as a promising tactic owing to its unique merits of high drug loading and has alleviated excipients‐associated toxicity.^[^ ^4^ ^]^ While self‐carrier drug systems still need poly(ethylene glycol)‐containing polymers for stealthy functions in vivo.^[^ ^5^ ^]^ It remains a big challenge to develop polymer‐free systems for drug delivery.^[^ ^6^ ^]^

Fabricating simple formulations by introducing clinically approved materials as drug delivery vectors is a straightforward approach and possesses prospect of potential clinical translation.^[^ ^7^ ^]^ Recently, a food and drug administration approved near‐infrared (NIR) dye, indocyanine green (ICG) was incorporated various delivery systems owing to the distinct advantages of good biocompatibility, desirable imaging of deep tissues,^[^ ^8^ ^]^ and non‐invasive phototherapy activity.^[^ ^9^ ^]^ Typically, ICG was co‐assembled with chemotherapeutics drugs,^[^ ^10^ ^]^ photosensitizers,^[^ ^11^ ^]^ and immuno‐modulators^[^ ^10^, ^12^ ^]^ for combination cancer therapy. However, the immature drug release and undesirable distribution in normal cells/tissues could inevitably result in potential systemic toxicity and limited efficacy.^[^ ^13^ ^]^

Implanting tumor microenvironment responsive repertoire is an effective strategy to realize precise treatment and simultaneously alleviate off‐target toxicity.^[^ ^14^ ^]^ Our group has reported a series of redox‐,^[^ ^5^, ^15^ ^]^ hypoxia‐,^[^ ^16^ ^]^ and enzyme^[^ ^17^ ^]^‐activatable PTX prodrug for improved chemotherapy. The construction of a PTX prodrug nanoparticle with the help of ICG would improve the drug delivery efficacy, chemotherapy selectivity, and NIR imaging of cancer.

**SCHEME 1:**
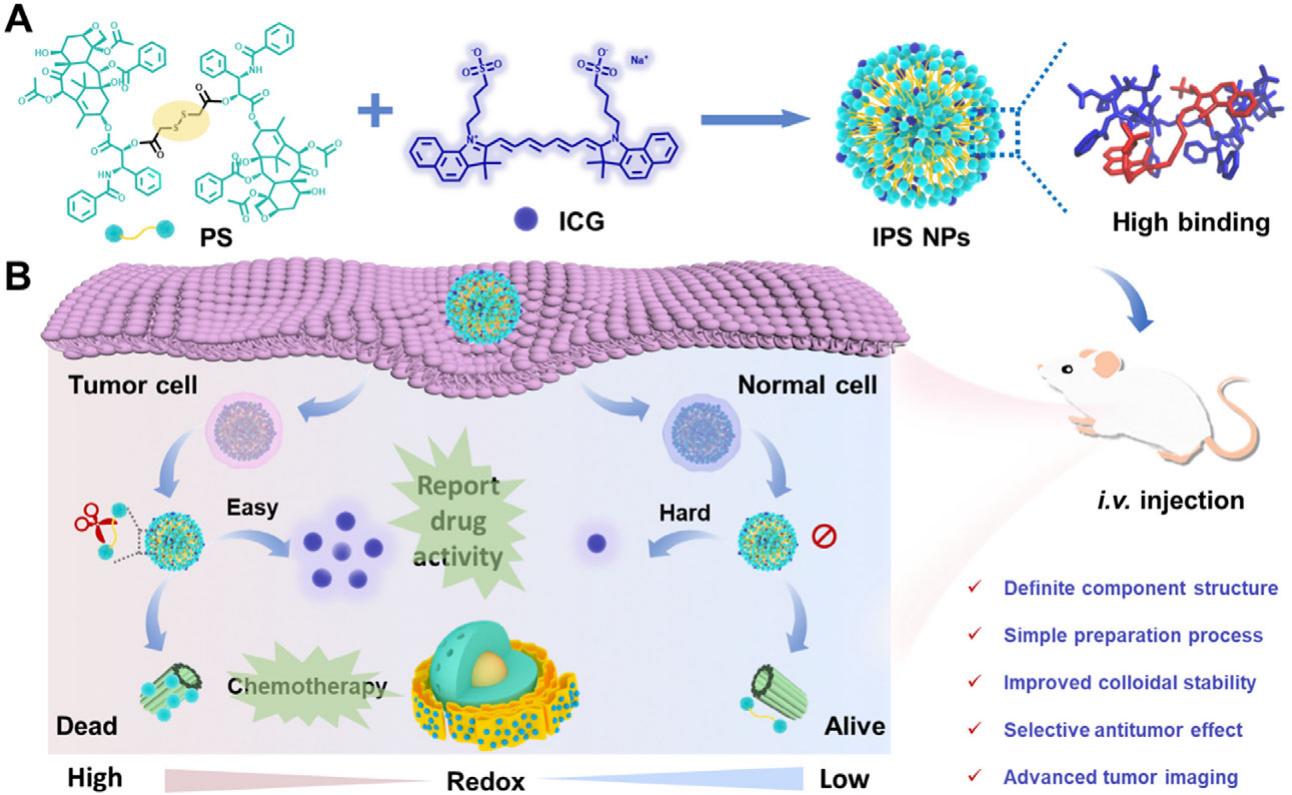
(A) Schematic illustration of the preparation of IPS NPs from the co‐assembly of ICG and PTX prodrug, and (B) the ICG‐potentiated prodrug delivery for tumor‐specific chemotherapy and real‐time therapeutic feedback

Herein, we report a prodrug delivery nanoplatform prepared from ICG‐driven co‐assembly with redox‐activatable PTX dimers for advanced tumor imaging and treatment. As shown in Scheme 1, the ICG could act as a surfactant to dissolve the PTX dimers bridged with 2,2′‐thiodiacetic acid (named as PS) in aqueous solutions to obtain two‐in‐one nanoparticles for drug delivery. The current formulation is able to integrate the following advantages: 1) facile preparation process, definite chemical structure, and composition ratio; 2) the robust colloidal stability because of high affinity between PTX prodrug and ICG; 3) the high drug content and controlled drug release; 4) tumor‐specific anticancer activity; 5) the NIR imaging capability of tumor and chemotherapy activity. We detailedly probe the ICG‐potentiated PTX prodrug nanoformulations and their potency of antitumor efficacy.

## 2 METHODS

### 2.1 Preparation of ICG potentiated PTX prodrug NPs

First, acetone solution (1 ml) of PS (1.74 mg) or PC (1.7 mg) was dropped into aqueous solution of ICG (1.25 mg, 2.5 ml) under vigorous agitation. After complete evaporation of the organic solvent, the mixture was centrifuged (5000 r/min, 5 min) to remove big aggregates and subsequently centrifuged (15,000 r/min, 15 min) again to remove the unbound ICG, and then two kinds of nanoparticles, namely IPS NPs and IPC NPs, respectively, were obtained by ultrasonic resuspension of the precipitation.

### 2.2 Preparation of BDP_2_‐C6/ICG NPs

First, *N*,*N*‐dimethylformamide solution (DMF, 1 ml) of BDP_2_‐C6 (0.8 mg) was dropped into aqueous solution of ICG (1.25 mg, 2.5 ml) under vigorous agitation. The big aggregates were removed by centrifugation (5000 r/min, 5 min). Then the supernatants containing organic solvent and unbound ICG were removed by centrifugation (15,000 r/min, 15 min). And the BDP_2_‐C6/ICG NPs were obtained by ultrasonic resuspension of the precipitation.

### 2.3 Preparation of TPP_2_‐C8/ICG NPs

First, DMF solution (1 ml) of TPP_2_‐C8 (1.4 mg) was dropped into aqueous solution of ICG (1.25 mg, 2.5 ml) under vigorous agitation. The big aggregates were removed by centrifugation (5000 r/min, 5 min). Then the supernatants containing organic solvent and unbound ICG were removed by centrifugation (15,000 r/min, 15 min). And the TPP_2_‐C8/ICG NPs were obtained by ultrasonic resuspension of the precipitation.

### 2.4 In vitro stability of NPs

The ICG‐inspired prodrug NPs were treated with different solutions, including 0.9% NaCl, 5% glucose, PBS (pH 7.4) containing 10% fetal bovine serum (FBS), and mouse plasma at 37°C for specific time. The dynamically changing sizes were measured by dynamic light scattering (DLS).

### 2.5 PTX release from IPS NPs and IPC NPs initiated by DTT and H_2_O_2_

The in vitro cleavage of bridged linkers and following PTX release from prodrug NPs were studied by incubating IPS NPs (PS, 50 µg) or IPC NPs (PC, 49 µg) in release solutions (0.5 ml, ethanol/water, V/V, 3/7) containing DTT (10 mM) or H_2_O_2_ (10 mM) at 37°C. At a predetermined post‐incubation time, 0.5 ml of fresh acetonitrile was added, and the obtained mixture was centrifuged (10,000 r/min, 10 min). Then we extracted the resulting supernatants and subsequently examined the drug release using HPLC (detection wavelength, 231 nm).

### 2.6 Molecular dynamic simulations

The initial models of PTX, PS, and ICG were built and optimized using Avogadro^[^ ^18^ ^]^ software. The molecular dynamic simulations were performed by using CHARMM36 2021 force field with the CGenFF parameters and GROMACS software (GROMACS‐5.1.5).^[^ ^19^ ^]^ We used periodic boundary conditions to put the corresponding initial models in a cubic box,^[^ ^20^ ^]^ and meanwhile TIP3P water model was used as the solvent. Using steepest descent algorithm,^[^ ^21^ ^]^ an initial energy was minimized, and 50 ns NPT ensemble simulation was carried out for data analysis. The GROMACS analysis tool *gmx_cluster* was used to discover typical clusters conformations present in the simulation trajectories. These conformations were used in following molecular docking to investigate the binding energy between two molecules (PTX and ICG, PS and ICG, PS and PS) which was predicted through AutoDock 4.2^[^ ^22^ ^]^ software. The Lamarckian genetic algorithm in AutoDock 4.2 was used to perform docking process, in which the number of genetic algorithm runs was 100, the maximum number of evaluations was 2,500,000 per run, and the population size was 300.

### 2.7 Statistical analysis

All data were analyzed with one‐way ANOVA test. The results were represented as mean ± standard deviation (SD) unless specified otherwise. n.s., no significance, *P < 0.05, **P < 0.01, ***P < 0.001.

## 3 RESULTS AND DISCUSSION

### 3.1 Preparation and characterization of ICG‐potentiated PTX prodrug NPs

**FIGURE 1:**
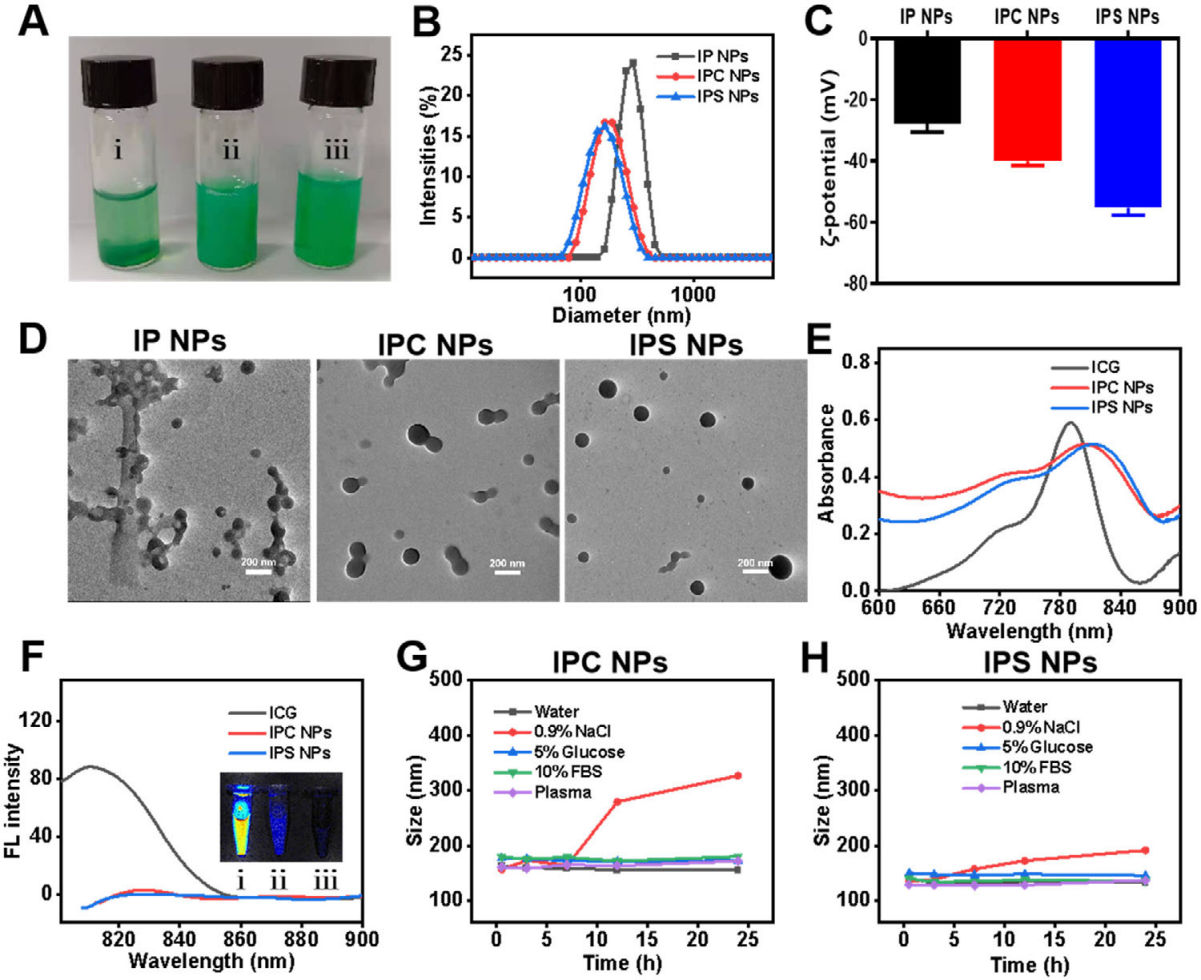
Characterization and photophysical property of ICG‐potentiated PTX prodrug NPs. (A) The picture of suspensions prepared from the coassembly of ICG with (i) PTX, (ii) PC, and (iii) PS. (B) Size distribution, (C) ζ‐potential, and (D) TEM images of IP NPs, IPC NPs, and IPS NPs. (E) The absorption spectra, (F) fluorescence emission spectra, and corresponding fluorescence images (from left to right, ICG, IPC NPs, and IPS NPs) of free ICG, IPC NPs, and IPS NPs with equivalent ICG concentration. Size changes of (G) IPC NPs and (H) IPS NPs in different incubation media, including water, 0.9% NaCl, 5% glucose, PBS containing 10% FBS, and mouse plasma

We first synthesized a PS using 2,2′‐dithiodiacetic acid as the bridged linker according to our previous work.^[^ ^15a^ ^]^ The PTX prodrug bridged with adipic acid was served as the nonresponsive control and abbreviated as PC. The ICG‐containing PTX prodrug nanoparticles were prepared using the nanoprecipitation method. In detail, the obtained PTX prodrugs (PS and PC) were dissolved in acetone and then dropped into ICG aqueous solution under vigorous agitation. Once the organic solvent was completely evaporated, the mixtures were subjected to centrifugation to remove unbound ICG, and then two kinds of nanoparticles were obtained, namely IPS NPs and IPC NPs, respectively. The assembly of pure PTX and ICG was also investigated for comparison. Similar to the previous work,^[^ ^10d^ ^]^ it is hard to obtain stable nanoparticles with high utilization rates of PTX and ICG using the same method to prepare ICG‐potentiated PTX prodrug NPs. Different from the assembly of PS/PC with ICG, the mixtures of PTX and ICG were prone to form the large aggregates (Figure 1A). The main reason for the formation of precipitates is the easy tendency of PTX to self‐aggregate and crystallize.^[^ ^23^ ^]^ After removing the formed precipitates and unbound ICG, the nanosuspensions containing PTX and ICG (IP NPs) were obtained with the average particle size of 220 nm as examined by DLS. In comparison, no precipitates were seen for IPS NPs and IPC NPs, which were smaller nanoscale particles with the hydrodynamic diameters of about 143 and 150 nm, respectively (Figure 1B). IPS NPs possessed the lowest negative potential of −55.2 mV, compared with that of −40 mV for IPC NPs and −27.7 mV for IP NPs (Figure 1C). The critical aggregate concentrations (CAC) of IPS NPs and IPC NPs are determined to be 6.15 and 6.56 µg ml^–1^, respectively (Figure S1). The bridged linkers of prodrugs acting as “structure defect” may break the long range‐ordered molecular arrangement and decrease the degree of crystallinity, thus increasing intermolecular interaction between PTX prodrug and ICG. IPS NPs and IPC NPs exhibited more uniformly monodispersed spherical morphology as confirmed by the transmission electron microscopy (TEM) images, while PS NPs and PC NPs tended to stick for each other (Figure 1D and Figure S2). The disulfide bond‐facilitating assembly may account for the smaller sizes and well‐dispersed morphology of IPS NPs.^[^ ^24^ ^]^ The absorption spectra of IPS NPs and IPC NPs had a significant redshift of about 20 nm compared to ICG (Figure 1E and Figure S3). In addition, the obviously quenched fluorescence of IPS NPs and IPC NPs demonstrated the successful assembly of ICG with PTX prodrugs (Figure 1F). After incubation with water, 5% glucose, PBS (pH = 7.4) containing 10% FBS, and mouse plasma for 24 h, the particle sizes of IPS NPs remained nearly unchanged, suggesting the robust colloidal stability (Figure 1H). The IPC NPs were kept stable in the above four solutions except 0.9% NaCl, accompanied by evidently increasing sizes (Figure 1G). The increase of particle size of IPS NPs in 0.9% NaCl was significantly less than that of IPC NPs, which was probably ascribed to the contribution of disulfide bond to the assembled stability. The IPS NPs could remain stable for 2 months as evidenced by the negligible size distribution change, guaranteeing the feasibility of long‐term storage (Figure S4). In addition, the particle sizes of self‐assembled PS NPs changed dramatically in 0.9% NaCl (Figure S5). These results confirmed that the assembly of ICG with PS was more stable than that with PC, revealing that the disulfide bonds contributed to the colloidal stability of the nanoparticles.

### 3.2 Assembly mechanism of ICG‐potentiated PTX prodrug NPs

**FIGURE 2:**
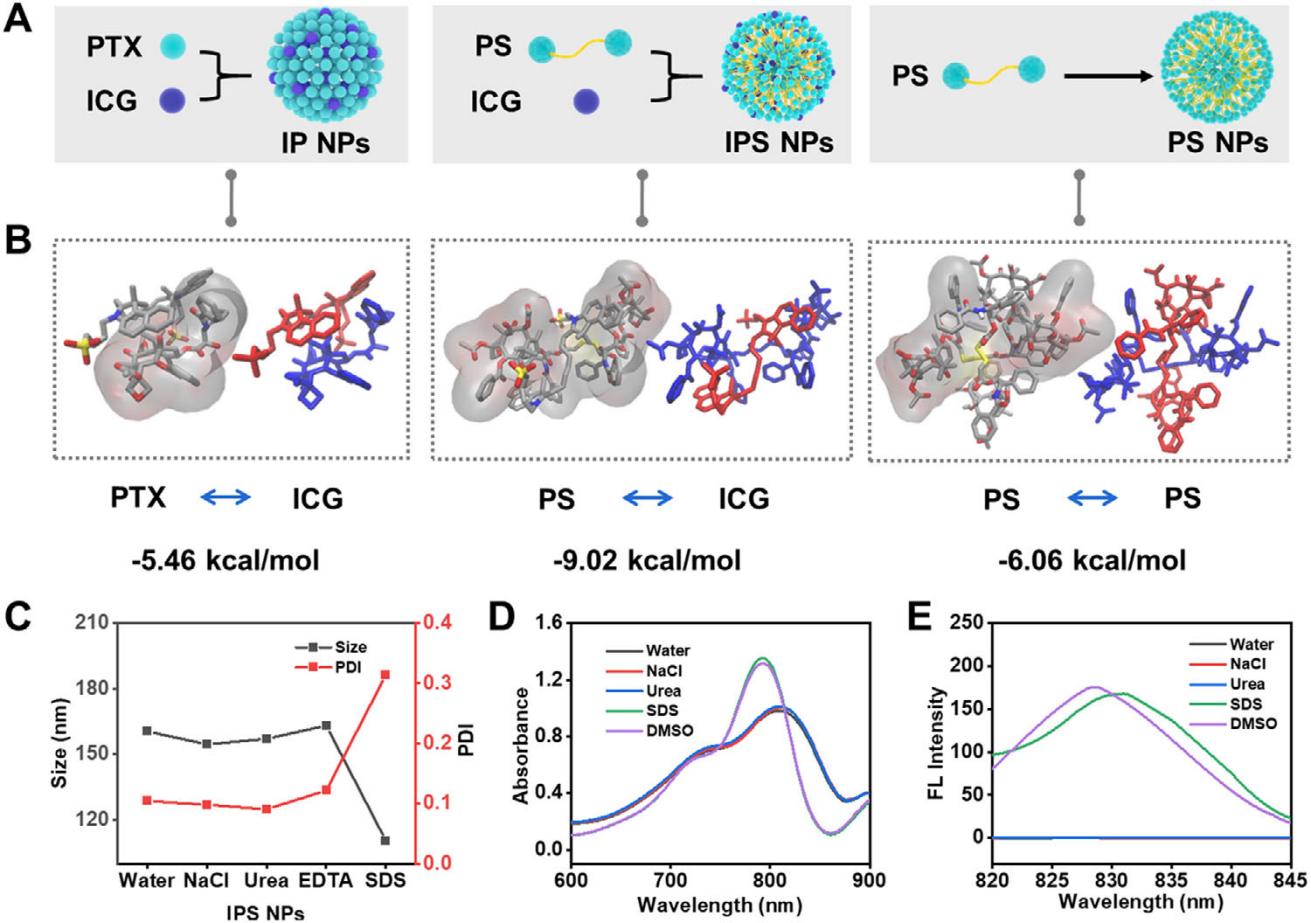
The assembly mechanism of ICG and PTX prodrugs. (A) Scheme illustration of assembly of the three nanoparticles (IP NPs, IPS NPs, and PS NPs). (B) The binding free energy calculated from molecular docking methods between the corresponding molecules (from up to down, PTX, and ICG, PS and ICG, PS and PS). (C) The particle size and PDI changes of IPS NPs after being incubated with NaCl, urea, EDTA, and SDS for 24 h. Changes in absorption (D) and fluorescence emission (E) spectra of IPS NPs in different treating solutions (water, NaCl, urea, SDS, and DMSO)

We use molecular docking methods to investigate the intermolecular interaction using the binding energy as key factors. The conformation used in docking is generated by all‐atom molecular dynamic simulations with CHARMM36 force field^[^ ^25^ ^]^ and GROMACS software.^[^ ^19^, ^26^ ^]^The binding energy is obtained by the sum of Vdw+Hbond+desolv (dispersion and repulsion, hydrogen bond, desolvation) energy, electrostatic energy, and torsional energy. The binding energies between PTX and ICG, PS and ICG, PS and PS are calculated to be −5.46, −9.02, and −6.06 kcal/mol, respectively (Figure 2A,B). According to the thermodynamic principle, the lower free energy (ΔG < 0) means more robust stability of nanomaterials. The lowest binding energy for PS and ICG can account for the more facile assembled behavior and stable colloidal stability of IPS NPs than that of IP NPs and PS NPs. We also calculate the bond angles of disulfide and dicarbide bond in the representative geometry of PS and PC, respectively. Compared with ─C─CC─C─ (113.22°/113.47°) for PC, the bond angles of ─C─SS─C─ (103.94°/103.68°) for PS is closer to 90°, suggesting improved structural flexibility for PS to interaction with ICG.^[^ ^5b^ ^]^ Besides, compared with the dihedral angles values of 166.33° for C─CC─C, the 90.20° for C─SS─C is closer to 90^o^, which suggests preferential conformation in favor of the assembly.^[^ ^27^ ^]^ Above results confirm the enhanced assembling behavior and colloidal stability of IPS NPs.

To explore the assembling mechanism of PTX dimers and ICG, IPS NPs and IPC NPs were treated with NaCl, urea, ethylenediaminetetraacetic acid disodium salt (EDTA), and sodium dodecyl sulfate (SDS).^[^ ^10c^ ^]^ The apparent changes in size and polydispersity index (PDI) of IPS NPs upon treatment with SDS implied that the hydrophobic interactions dominated the assembly process (Figure 2C). After incubation with NaCl and urea, the maximum absorption peak of IPS NPs exhibited ignorable changes (Figure 2D). Meanwhile, the absorption spectra of IPS NPs had a blueshift in the presence of SDS and dimethyl sulfoxide (DMSO), which was ascribed to the disintegration of IPS NPs. Consistently, the fluorescence was recovered upon incubation with SDS and DMSO (Figure 2E). IPC NPs had similar changes as IPS NPs, accompanied by increased size and PDI, recovery of absorption, and fluorescence spectra (Figure S6). These results confirmed that hydrophobic interaction was the primary driving force for assembly of PTX prodrugs and ICG, which is consistent with the theoretical calculations.

### 3.3 Redox responsive drug release from ICG‐potentiated PTX prodrug NPs

**FIGURE 3:**
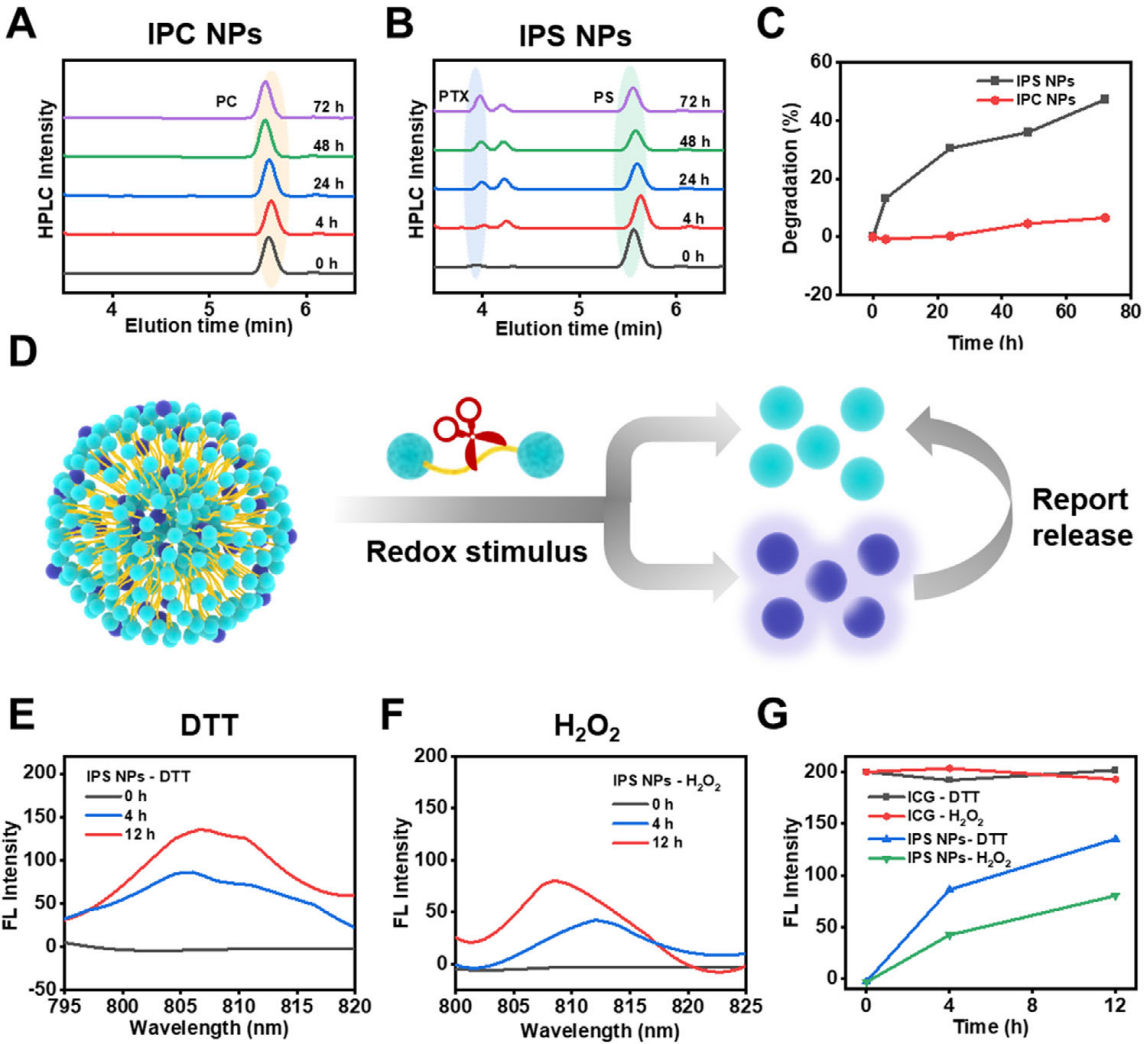
The redox responsiveness of ICG‐potentiated PTX prodrug NPs. Reduction responsiveness of (A) IPC NPs and (B) IPS NPs upon treatment with 10 mM DTT as monitored by HPLC. (C) The degradation of IPC NPs and IPS NPs treated with 10 mM DTT. (D) The corresponding schematic illustration of drug release from IPS NPs and accompanied fluorescence recovery triggered by redox stimulus. Fluorescence emission spectra of IPS NPs treated with (E) 10 mM DTT and (F) 10 mM H_2_O_2_. (G) The change rates of fluorescence intensity of ICG and IPS NPs at about 810 nm with equivalent ICG concentration upon incubation with DTT and H_2_O_2_

To evaluate the redox responsiveness of IPS NPs and IPC NPs, we used dithiothreitol (DTT) and hydrogen peroxide (H_2_O_2_) to monitor the drug release by high performance liquid chromatograph (HPLC). The disulfide bond of PS in IPS NPs can be cleaved to form intermediates in 4 h, which gradually transformed into PTX (Figure 3B). Finally, the prodrug decomposed 47% at 72 h post treatment (Figure 3C). Relatively, IPC NPs can hardly release active PTX after being treated with 10 mM DTT (Figure 3A). Then we studied their oxidant responsiveness using 10 mM H_2_O_2_. After incubation with H_2_O_2_ for 72 h, IPS NPs exhibited more sensitive release of PTX compared with IPC NPs (Figure S7). The detailed redox‐triggered release mechanism was clarified according to our previous work.^[^ ^15a,15d^ ^]^ The PTX release from IPS NPs in redox condition is faster than that from IPC NPs. Owing to the poor binding efficacy of PTX and ICG, the PTX release from IPS NPs could facilitate the disassembly of nanostructure, thus recovering the fluorescence of ICG (Figure 3D). After incubation with DTT and H_2_O_2_, the fluorescence of IPS NPs was gradually enhanced in 12 h (Figure 3E–G). It is noted that the fluorescence intensity of IPS NPs did not recover completely, which may be ascribed to the inadequate incubation time of IPS NPs with DTT and H_2_O_2_ for entire release of PTX. This controlled “off‐on” fluorescence signal could be used to not only image tumor regions, but also real‐time reflect the disintegration of IPS NPs.^[^ ^28^ ^]^

### 3.4 Cellular internalization of ICG‐potentiated PTX prodrug NPs

**FIGURE 4:**
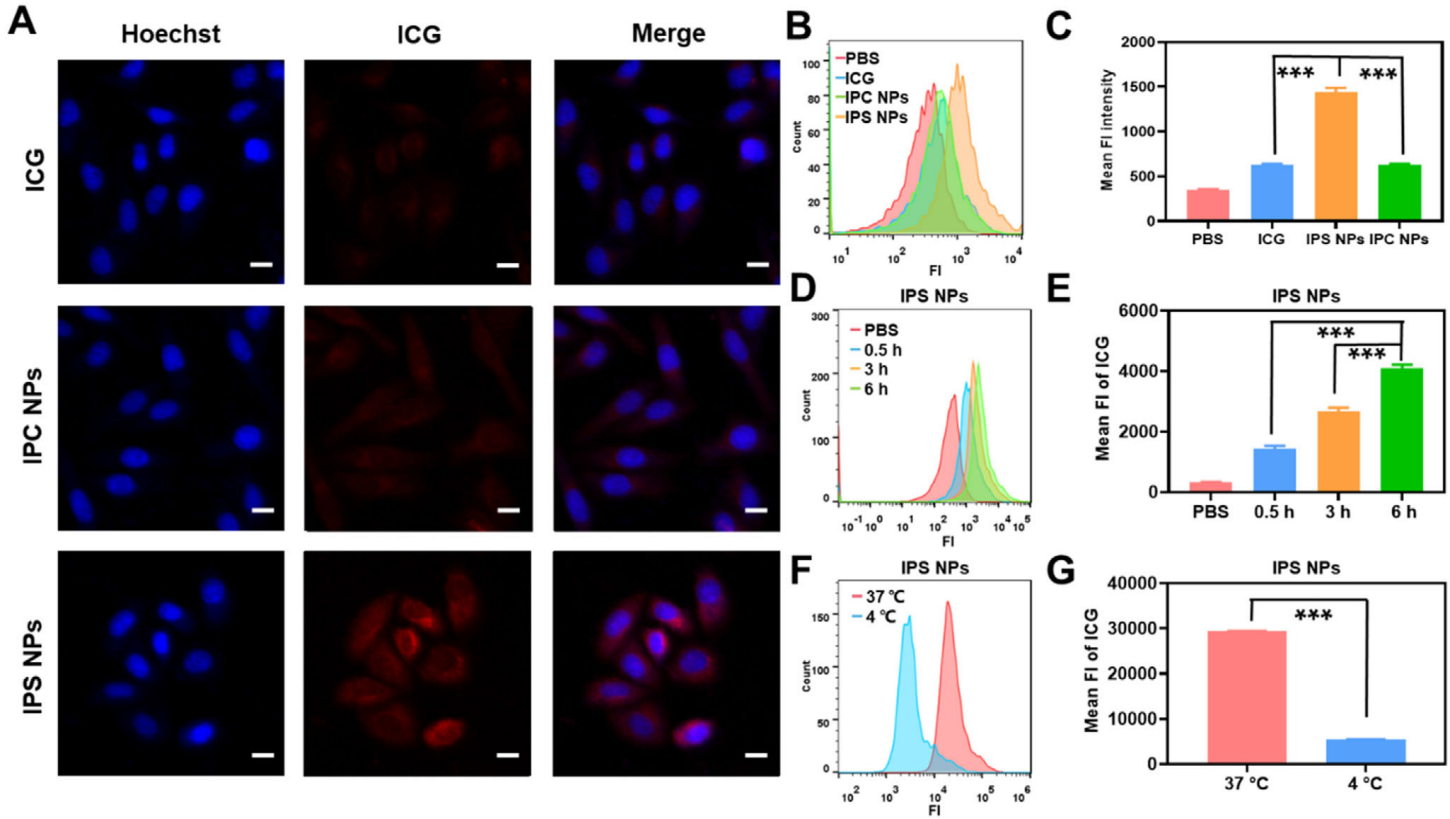
Cellular internalization of ICG‐potentiated PTX prodrug NPs. (A) CLSM images and (B) FCM analysis of HeLa cells treated with ICG, IPS NPs, and IPC NPs. Scale bars, 20 µm. (D) Quantitative analysis of endocytosis of IPS NPs as a function of incubation time by FCM. (F) Quantitative analysis of the endocytosis difference of IPS NPs at 4°C and 37°C by FCM. (C,E,G) The mean fluorescence intensity of ICG‐potentiated PTX prodrug NPs by FCM in different incubation conditions corresponding to (B), (D), and (F), respectively. n.s., no significance, *P < 0.05, **P < 0.01, and ***P < 0.001

We next investigated the endocytosis of free ICG and PTX prodrug NPs by confocal laser scanning microscopy (CLSM). We incubated HeLa cells with ICG, IPS NPs, and IPC NPs at 37°C. As shown in Figure 4A, the effective cellular internalization of IPS NPs by cancer cells was verified by the obvious fluorescence at the cytoplasm. And the intracellular red fluorescence of IPS NPs was higher than that of IPC NPs and ICG as determined by flow cytometry (FCM), indicating the intracellular prodrug activation of IPS NPs (Figure 4B,C). The intracellular fluorescence intensity enhanced as the incubation time increased from 0.5 to 6 h, suggesting that cellular endocytosis of IPS NPs proceeds in a time‐dependent manner (Figure 4D,E, and Figure S8). In addition, the fluorescence intensity at 4°C was much lower than that at 37°C, verifying the energy‐dependent intracellular endocytosis (Figure 4F,G, and Figure S9).

### 3.5 Cytotoxicity of ICG‐potentiated PTX prodrug NPs

**FIGURE 5:**
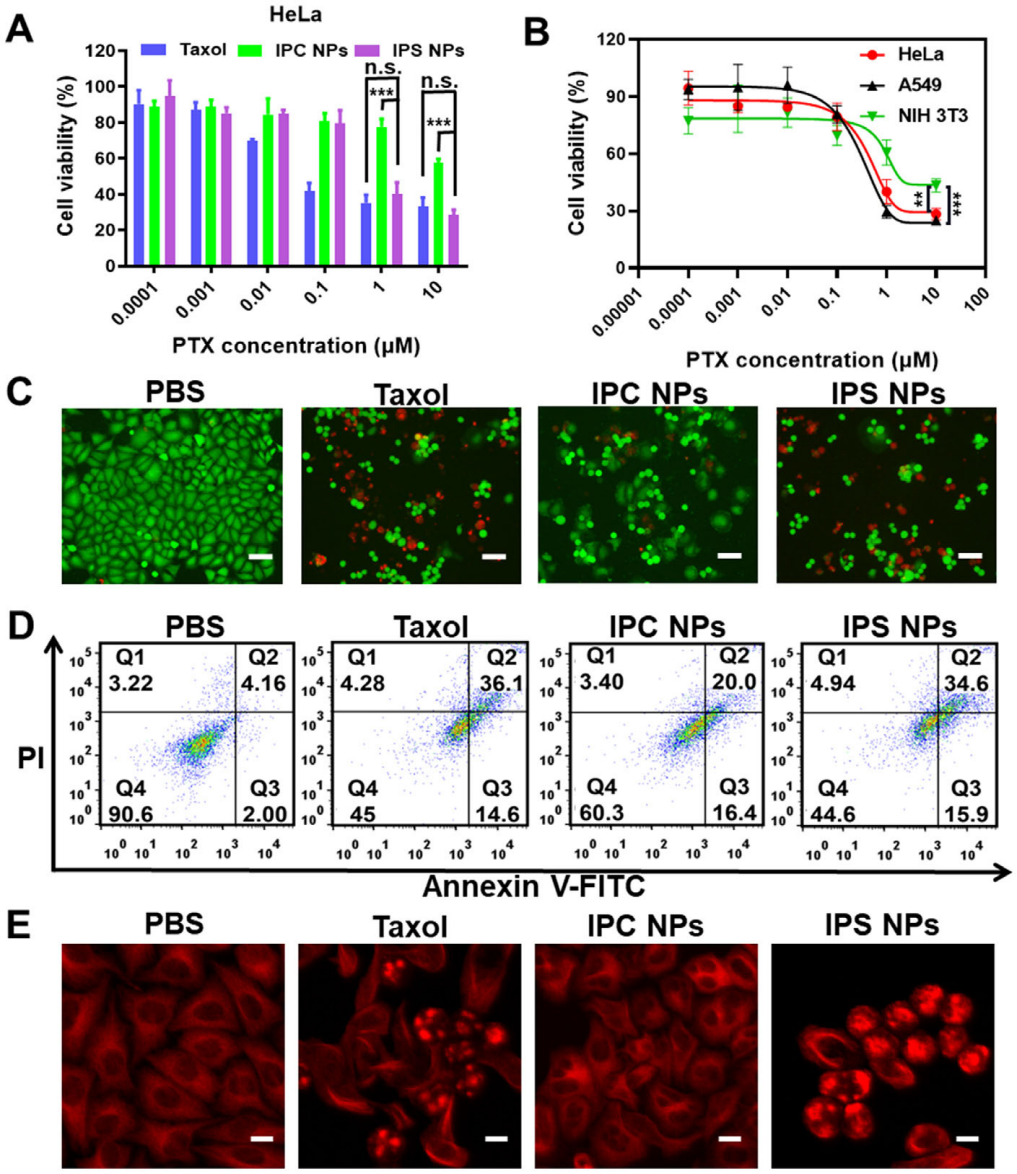
Selective cytotoxic effects of IPS NPs toward cancer cells. (A) Cell viability of Taxol, IPC NPs, and IPS NPs (PTX concentration: 10^–4^–10 µM) toward HeLa cells for 48 h. (B) Cytotoxicity of IPS NPs toward cells with different levels of redox species (High, A549 and HeLa; Low, NIH 3T3) for 48 h. (C) Live‐dead stained fluorescence images and (D) FCM quantification of apoptosis and necrosis of HeLa cells incubated with Taxol, IPC NPs, and IPS NPs (PTX: 10 µM) for 48 h. (E) CLSM images of microtubules staining of HeLa cells cultured with Taxol, IPC NPs, and IPS NPs (PTX: 10 µM) for 24 h. Scale bars, (C) 100 µm and (E) 20 µm. n.s., no significance, *P < 0.05, **P < 0.01, and ***P < 0.001

3‐(4,5‐dimethylthiazol‐2‐yl)‐2,5‐diphenyl‐tetrazolium bromide (MTT) assays were conducted to evaluate the in vitro tumor inhibition effect of ICG‐anchored PTX prodrug NPs. We first confirmed the good compatibility of ICG (Figure S10). IPS NPs exhibited a concentration‐dependent cytotoxicity toward HeLa and A549 cells (Figure 5A and Figure S11A). At the concentration of 10 µM of PTX, the cytotoxicity of IPS NPs was more potent than that of IPC NPs, and the cell survival rates were 28% and 57%, respectively. The enhanced cytotoxicity of IPS NPs was attributed to intracellularly redox‐triggered drug release. To explore the effect of intracellular redox microenvironment on prodrug activation and subsequent cytotoxicity, we further investigated the killing efficacy of IPS NPs and IPC NPs to NIH 3T3 cells, which contain a low level of redox species. Obviously, the cell killing capability of IPS NPs was reduced at the same PTX concentration (Figure 5B and Figure S11B). The IC_50_ values of IPS NPs against A549, HeLa, and NIH 3T3 cells are calculated to be 0.30, 0.47, and 3.85 µM, respectively. The results indicated that IPS NPs possessed the selective cytotoxicity in part toward cancer cells, compared with normal cells. The enhanced cytotoxicity compared with nonresponsive IPC NPs toward cancer cells and reduced cytotoxicity toward normal cells indicated that IPS NPs possessed superiorly selective chemotherapy activity. Then we performed the live‐dead staining experiments against HeLa cells to demonstrate the cytotoxicity difference of the two NPs. As shown in Figure 5C, IPS NPs possessed more dead cells with red fluorescence compared with IPC NPs group. We also carried out the crystal violet staining experiment to verify the superior antitumor activity of IPS NPs. Crystal violet is an alkaline dye that prefers to bind to the endonuclear DNA, thus resulting in the purple cells. Fewer purple cellular nuclei were seen after incubation with IPS NPs, relative to IPC NPs (Figure S12). Next, we used annexin V‐FITC/PI co‐staining method to quantitatively analyze cell apoptosis initiated by PTX prodrug NPs. The similar total (early and late) cell apoptosis rates of IPS NPs (50.5%) as Taxol (50.7%) confirmed their comparable anticancer activity, which was higher than that of IPC NPs (36.4%) (Figure 5D). In order to evaluate the detailed anticancer mechanism, we performed a tubulin immunostaining experiment and monitored the changes of the microtubule morphology. The microtubules of PBS group were well organized, and obvious tubulin clusters emerged in the cells treated with IPS NPs, suggesting their sufficient PTX release (Figure 5E). In comparison, only mitotic disorders emerged in the IPC NPs group.

### 3.6 In vivo biodistribution of ICG‐potentiated PTX prodrug NPs

**FIGURE 6:**
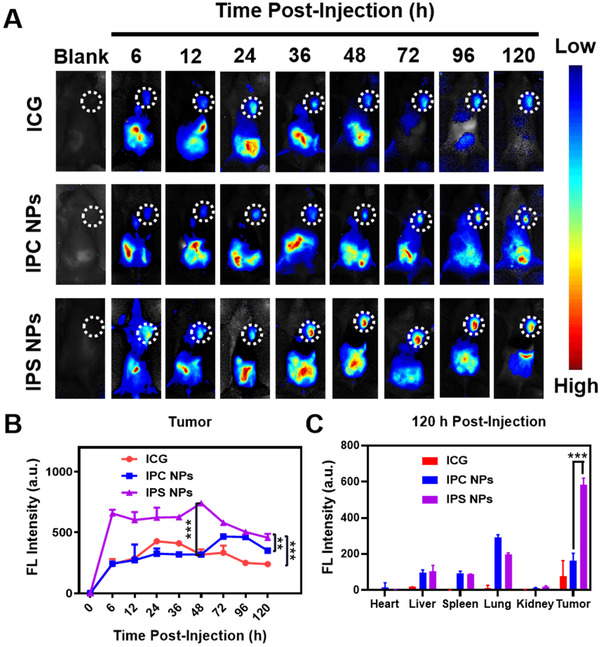
The biodistribution of ICG‐potentiated PTX prodrug NPs. (A) The real‐time NIR imaging images of mice with xenograft 4T1 tumor after systematic injection of ICG, IPC NPs, and IPS NPs. (B) Semi‐quantitation of intratumoral fluorescence changes of ICG, IPC NPs, and IPS NPs groups as the post‐injection time increased. (C) The semi‐quantitation of fluorescence intensity of excised major organs and tumor of different groups. n.s., no significance, *P < 0.05, **P < 0.01, and ***P < 0.001

The biodistribution of PTX prodrug NPs was studied using 4T1 tumors‐bearing mice. The mice with the tumor sizes at ∼200–300 mm^3^ were intravenously administrated with ICG, IPS NPs, and IPC NPs at identical ICG dose. The real‐time fluorescence images of mice were collected through in vivo imaging system. The mice were sacrificed and tumor/major organs were collected to examine the distribution of NPs at 120 h post‐treatment. The fluorescence signal of IPS NPs reached maximum at 6 h, and remained at a relatively high value until 48 h post‐injection. Compared with ICG and IPC NPs group, the intratumoral fluorescence intensity of IPS NPs group was much stronger, manifesting that IPS NPs possibly possessed enhanced tumor accumulation and prodrug activation (Figure 6A). The semi‐quantitative intratumorous fluorescence curve further confirmed the above results (Figure 6B). The strongest fluorescence of the excised tumor in IPS NPs group was well consistent with the intratumorous curve (Figure 6C). We speculated that superior colloidal stability may account for the preferential tumor accumulation and imaging.

### 3.7 In vivo antitumor effect of ICG‐potentiated PTX prodrug NPs

**FIGURE 7:**
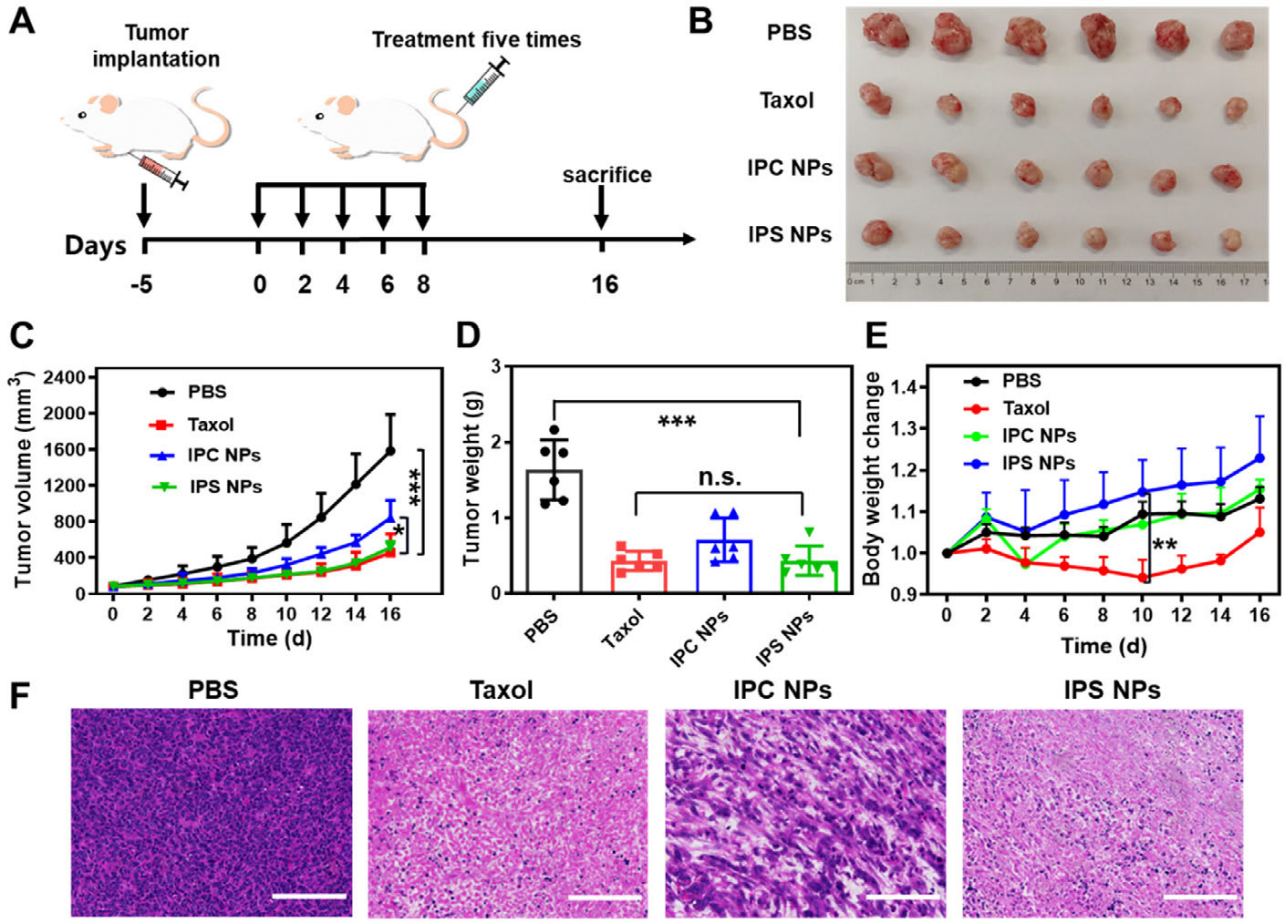
In vivo antitumor effect. (A) The procedure of animal experimental treatment. (C) The growing curve of tumor volume of 4T1‐bearing mice after being intravenously administrated with Taxol, IPS NPs, and IPC NPs at identical PTX dose (15 mg/kg, *n* = 6). (B) Pictures and (D) weights of isolated tumors in different treatment groups. (E) The body weight change (W_t_/W_0_) of 4T1 tumor‐suffering mice after being treated with PBS, Taxol, IPC NPs, and IPS NPs. (F) H&E histological stained images of isolated tumor slices of above four groups. Scale bars, 100 µm. n.s., no significance, *P < 0.05, **P < 0.01, and ***P < 0.001

We used a xenograft 4T1 tumor model to examine the antitumor effects of ICG‐anchored PTX prodrug NPs. The mice with the tumor size reaching ∼50–100 mm^3^ were stochastically divided into four groups with different treatments: PBS, Taxol, IPS NPs, and IPC NPs. The animal experiment proceeded as the program illustrated in Figure 7A. At 5 days post‐implanted of tumor, mice were systemically administrated with Taxol and the PTX prodrug NPs at identical PTX dose (15 mg/kg). The administrations were continued every other day for 5 times. The tumor volume in the PBS group increased very rapidly, while the Taxol and IPS NPs treatments exhibited extremely retardant tumor growth (Figure 7C). It can be seen that IPS NPs were more potent than that of IPC NPs in inhibition of tumor growth, and comparable with Taxol, which may be ascribed to the improved tumor accumulation and continuous drug release at tumor sites. The pictures and weights of excised tumors agreed well with the tumor size growing profiles (Figure 7B,D). The fewest tumor cells, lowest cellular integrity, most cellular nuclear ablation, and karyopyknosis were exhibited in the histological H&E stained images of tumor slices in Figure 7F, which further confirmed their effective antitumor performance of IPS NPs. We next evaluated the biosafety of Taxol and ICG‐anchored PTX prodrug NPs by monitoring body weight changes and routine blood tests. Mice in IPS NPs and IPC NPs groups showed sustainable increase of body weight during the treatment, indicating their low systemic toxicity (Figure 7E). In comparison, Taxol groups exhibited obvious body weight loss after the systemic administration. The parameters of routine blood test in IPS NPs and IPC NPs groups were similar to those in PBS group within the normal range, indicating that ICG enhanced PTX prodrug NPs had no hematopoietic dysfunction (Figure S13A). H&E staining images of heart, liver, spleen, lung, and kidney exhibited no distinct histological damage (Figure S13B). These results manifested that ICG‐containing PTX prodrug NPs possess reduced off‐target toxicity. Therefore, IPS NPs were identified as a promising cancer treatment candidate with comparable antitumor outcome but reduced systemic toxicity with commercial Taxol.

### 3.8 The universality of ICG‐potentiated strategy

**FIGURE 8:**
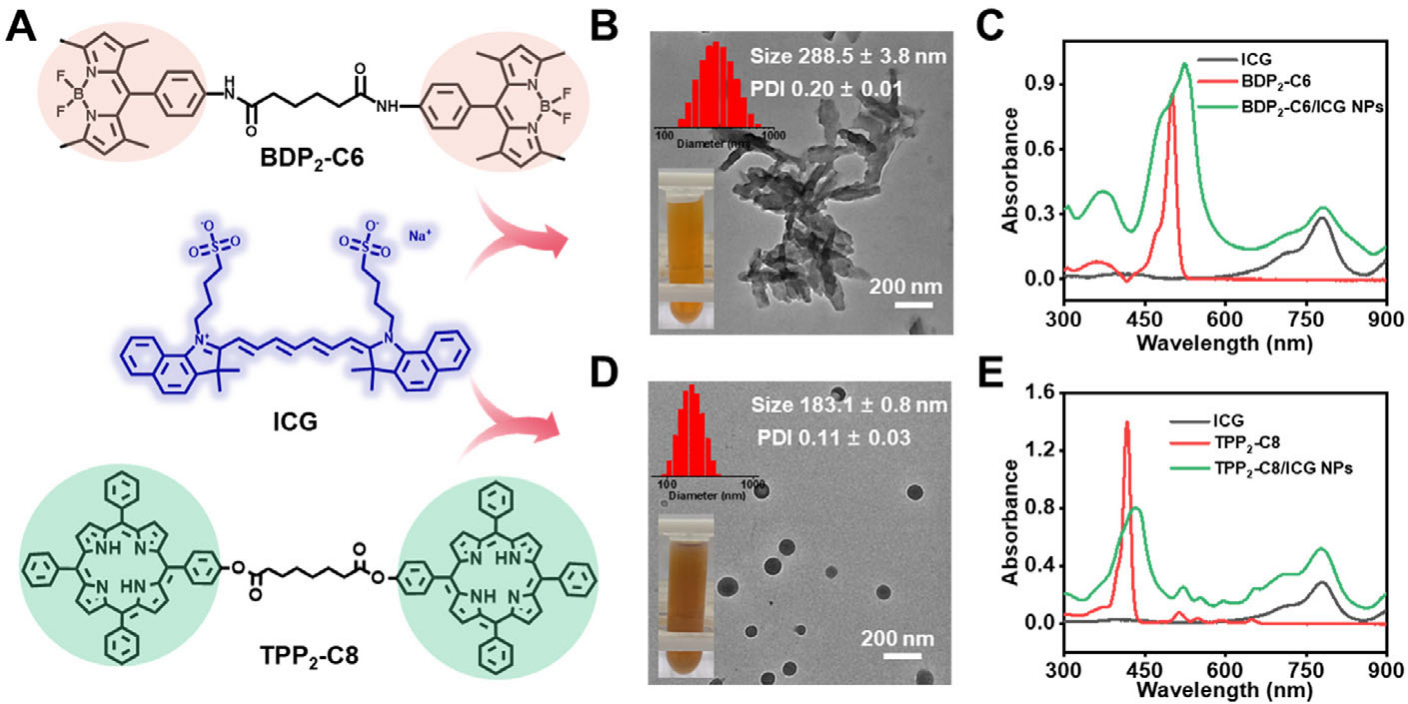
The universality of ICG‐potentiated assembly of hydrophobic BODIPY and porphyrin dimer. (A) The structure of BDP_2_‐C6 and TPP_2_‐C8. The size (top left inset), picture (down left inset), and the corresponding TEM image of (B) BDP_2_‐C6/ICG NPs and (D) TPP_2_‐C8/ICG NPs. (C) The absorption spectra of ICG in water, BDP_2_‐C6 in DMF, and BDP_2_‐C6/ICG NPs. (E) The absorption spectra of ICG in water, TPP_2_‐C8 in DMF, and TPP_2_‐C8/ICG NPs

Inspired by the superior performance of ICG‐facilitated assembly and drug delivery, we explored the versatility of assembling ICG with other hydrophobic molecules. The representative photosensitizer porphyrin and fluorescent dyes dipyrromethene boron difluoride (BODIPY) were chosen as the model molecules. The TPP_2_‐C8 was synthesized according to the previous work by our group.^[^ ^29^ ^]^ The accurate synthesis of BDP_2_‐C6 was confirmed by the ^1^H NMR spectrum (Figure 8A and Figure S14). Similar with the assembly results of IPS NPs, BDP_2_‐C6 or TPP_2_‐C8 in combination with ICG formed smaller nanoparticles, as evidenced by the diameter of 288.5 nm for BDP_2_‐C6/ICG NPs and 183.1 nm for TPP_2_‐C8/ICG NPs (Figure 8B,D). Compared with free molecule in organic solvent, the obvious bathochromic‐shift of absorption of BDP_2_‐C6 and TPP_2_‐C8 in NPs confirmed their assembled states (Figure 8C,E). Compared with BDP_2_‐C6, TPP_2_‐C8 and ICG, the fluorescence was completely quenched for BDP_2_‐C6/ICG NPs and TPP_2_‐C8/ICG NPs, suggesting the successful coassembly of ICG with BDP_2_‐C6 or TPP_2_‐C8 (Figure S15). This ICG‐potentiated assembly from drugs to photosensitizers and fluorescent dyes not only provides an alternative method to form nanoparticles, but also integrates various functionalized molecules into one system.

## 4 CONCLUSIONS

In summary, we developed ICG‐potentiated PTX prodrug nanoparticles for advanced NIR imaging and chemotherapy. This simple nanoparticle formulation possesses the precise molecular structure without addition of surfactants or auxiliaries. There is no carrier‐associated long‐term toxicity and immunogenicity. The hydrophilic nature of ICG endowed PTX prodrugs enhanced assembling capacity and colloidal stability as well as tumor bioimaging and precise treatment. The IPS NPs exhibited preferential tumor accumulation and comparable anti‐cancer effects but alleviated systemic toxicity with Taxol. We believe that ICG‐potentiated PTX prodrug nanoparticles have great potential in clinical tumor treatment. More interestingly, this ICG‐potentiated tactic can be expanded to other organic dimers, convincing us that this work provides a universal approach to improve drug delivery.

## CONFLICT OF INTEREST

The authors declare no conflict of interest.

## ETHICS STATEMENT

All animal experiments have been approved (Approved No. 20210033) by the Animal Welfare and Ethics Committee of Changchun Institute of Applied Chemistry, Chinese Academy of Sciences.

## Supporting information



Figure S1. The critical aggregate concentration of (A) IPS NPs and (B) IPC NPs.
Figure S2. The TEM images and size distribution (top left inset) of (A) PC NPs and (B) PS NPs.
Figure S3. The absorption spectra of (A) IPC NPs and (B) IPS NPs obtained by using different organic solvents (ethanol, acetone, and tetrahydrofuran).
Figure S4. The size distribution of IPS NPs after storage at room temperature for 2 months.
Figure S5. (A) Size and (B) PDI changes of PS NPs after incubation with water, 0.9% NaCl, 5% glucose, and PBS (pH 7.4) containing 10% FBS for 24 h.
Figure S6. (A) Changes in size and PDI of IPC NPs treated with water, NaCl, urea, EDTA, and SDS for 24 h.
Figure S7. Oxidation responsiveness of (A) IPC NPs and (B) IPS NPs upon incubation with 10 mM H_2_O_2_ as determined by HPLC
Figure S8. CLSM images of HeLa cells incubated with IPS NPs at different times. Scale bars, 20 µm.
Figure S9. FCM fluorescence quantification of endocytosis of IPC NPs at 4°C and 37°C.
Figure S10. In vitro cytotoxicity of ICG against HeLa cells for 24 h.
Figure S11. In vitro cytotoxicity of Taxol, IPC NPs, and IPS NPs against (A) A549 cells and (B) NIH 3T3 for 48 h.
Figure S12. Crystal violet staining pictures of HeLa cells incubated with PBS, Taxol, IPC NPs, and IPS NPs at equivalent PTX concentration of 10 µM for 48 h. Scale bars, 100 µm.
Figure S13. (A) Routine blood analysis of mice in PBS, Taxol, IPC NPs, and IPS NPs group.
Figure S14. ^1^H NMR spectrum of BDP_2_‐C6 in DMSO‐d6.
Figure S15. (A) Fluorescence emission spectra of BDP fluorophore in BDP_2_‐C6 (DMF), and BDP_2_‐C6/ICG NPs (water).

## Data Availability

All data required to reproduce these findings are included in the article and in the Supporting Information.
