# Supplementary material for: Indocyanine green potentiated paclitaxel nanoprodrugs for imaging and chemotherapy
Source: Exploration (Beijing). 2022 Jun 4;2(4):20220008. doi: 10.1002/EXP.20220008 (PMC10190853; doi:10.1002/EXP.20220008)
Supplement: Supplementary file 1 — Figure S1. The critical aggregate concentration of (A) IPS NPs and (B) IPC NPs. Figure S2. The TEM images and size distribution (top left inset) of (A) PC NPs and (B) PS NPs. Figure S3. The absorption spectra of (A) IPC NPs and (B) IPS NPs obtained by using different organic solvents (ethanol, acetone, and tetrahydrofuran). Figure S4. The size distribution of IPS NPs after storage at room temperature for 2 months. Figure S5. (A) Size and (B) PDI changes of PS NPs after incubation with water, 0.9% NaCl, 5% glucose, and PBS (pH 7.4) containing 10% FBS for 24 h. Figure S6. (A) Changes in size and PDI of IPC NPs treated with water, NaCl, urea, EDTA, and SDS for 24 h. Figure S7. Oxidation responsiveness of (A) IPC NPs and (B) IPS NPs upon incubation with 10 mM H2O2 as determined by HPLC Figure S8. CLSM images of HeLa cells incubated with IPS NPs at different times. Scale bars, 20 µm. Figure S9. FCM fluorescence quantification of endocytosis of IPC NPs at 4°C and 37°C. Figure S10. In vitro cytotoxicity of ICG against HeLa cells for 24 h. Figure S11. In vitro cytotoxicity of Taxol, IPC NPs, and IPS NPs against (A) A549 cells and (B) NIH 3T3 for 48 h. Figure S12. Crystal violet staining pictures of HeLa cells incubated with PBS, Taxol, IPC NPs, and IPS NPs at equivalent PTX concentration of 10 µM for 48 h. Scale bars, 100 µm. Figure S13. (A) Routine blood analysis of mice in PBS, Taxol, IPC NPs, and IPS NPs group. Figure S14. 1H NMR spectrum of BDP2‐C6 in DMSO‐d6. Figure S15. (A) Fluorescence emission spectra of BDP fluorophore in BDP2‐C6 (DMF), and BDP2‐C6/ICG NPs (water). [file EXP2-2-20220008-s001.docx]

Supporting Information

**Indocyanine Green Potentiated Paclitaxel Nanoprodrugs for Imaging and Chemotherapy**

*Xiujuan Xiang,^†,‡^ Xuan Feng,^†,‡^ Shaojin Lu,^†,‡^ Bowen Jiang,^†,‡^ Dengyuan Hao,^†,‡^ Qing Pei,*^,†^ Zhigang Xie*^,†,‡^ and Xiabin Jing^†^*

^†^State Key Laboratory of Polymer Physics and Chemistry, Changchun Institute of Applied Chemistry, Chinese Academy of Sciences, Changchun, Jilin 130022, P. R. China

^‡^University of Science and Technology of China, Hefei, Anhui 230026, P. R. China

***Email: xiez@ciac.ac.cn; peiqing@ciac.ac.cn

**Contents:**

**1. Materials and characterization**

**2. Methods**

**2.1. Synthesis of PTX/TPP dimers**

**2.2. Synthesis of BDP_2_-C6**

**2.3. Cellular uptake and cytotoxicity experiments**

**2.4. Animal experiments**

**3. Supporting Figures**

**1. Materials and characterization**

PTX and ICG were purchased from Dalian Meilun Biotechnology Co., Ltd.. 1-Ethyl-3-(3-dimethylaminopropyl) carbodiimide hydrochloride (EDC·HCl) was purchased from Shanghai yuanye Bio-Technology Co., Ltd.. 4-dimethylaminopyridine (DMAP), 2, 2'-thiodiacetic acid and adipic acid were purchased from Shanghai Aladdin Biochemical Technology Co., Ltd.. Octanedioic acid (9 ding chem) was used as received. Pyridine was purchased from Sarn Chemical Technology Co., Ltd.. Chloroform-d (CDCl3) was purchased from Qingdao Tenglong Weibo Technology Co., Ltd.. Methylrosanilnium chloride solution was purchased from Hebei Jianning Pharmaceutical Co., Ltd.. Annexin V-FITC/PI double staining cell apoptosis detection kit were obtained from Jiangsu KeyGEN Biotechnology Co., Ltd.. Calcein/PI Live/Dead Viability Assay Kit was purchased from Beyotime Biotechnology. Cell culture dishes were purchased from Guangzhou Jet Bio-Filtration Co., Ltd.. Analytical balance (XS105DU) and Rainin Pipettes from METTLER TOLEDO were used to quantify solid and liquid respectively.

**2. Methods**

**2.1. Synthesis of PTX/TPP dimers**

PTX dimers bridged with 2, 2'-thiodiacetic acid and adipic acid, abbreviated as PS and PC, were synthesized according to our previous work.^[1]^ TPP_2_-C8 was synthesized according to the work reported by our group.^[2]^

**2.2. Synthesis of BDP_2_-C6**

The 4,4-difluoro-8-(4-aminophenyl)-3,5-dimethyl-4-bora-3a,4a-diaza-s-indacene (BDP-N was synthesized as the method reported by the previous work.^[3]^ BDP_2_-C6 was synthesized through the amidation reaction of adipic acid and BDP-N. In detail, BDP-N (33.9 mg, 0.1 mmol) and adipic acid (5.8 mg, 0.04 mmol) was dissolved in dichloromethane (CH_2_Cl_2_), then EDC·HCl (150 mg, 0.78 mmol) and pyridine (6.3 mg, 0.08 mmol) were sequentially added. The reaction was stirred at ambient temperature for 24 h. The product was purified using silica gel column chromatography. The orange-yellow solid (5 mg) was obtained with yields of 16%.

**2.3. Cellular uptake and cytotoxicity experiments**

Similar cellular experimental means and installations were reported by our previous work.^[4]^ Briefly, confocal laser scanning microscope (CLSM) and flow cytometer (FCM) were used to investigate the cellular uptake of ICG, IPC NPs and IPS NPs. The cytotoxicity of ICG-potentiated PTX prodrug NPs was examined via MTT protocols. The calcein-AM/PI staining tests, crystal violet staining tests, cell apoptosis and necrosis detection assays and the immunostaining of tubulin tests were also performed to examine the potent antitumor efficacy of ICG-potentiated PTX prodrug NPs, and the incubation concentration was set as 10 μM of equivalent PTX.

**2.4. Animal experiments**

All animal experiments have been approved (Approved No. 20210033) by the Animal Welfare and Ethics Committee of Changchun Institute of Applied Chemistry, Chinese Academy of Sciences, and carried out according to the Regulations on the Administration of Experimental Animals formulated by the State Science and Technology Commission of the People's Republic of China and the Guiding Opinions on The Good Treatment of Experimental Animals issued by the Ministry of Science and Technology of the People's Republic of China. We used female BALB/c mice (20-25 g) with subcutaneous 4T1 xenograft tumor to investigate the biodistribution of ICG-potentiated PTX prodrug NPs. The tumor-suffering mice were intravenously injected with ICG, IPC NPs and IPS NPs at equivalent ICG dose of 10 mg/kg (drug weight/body weight), and the *in vivo* near-infrared (NIR) fluorescence images were collected by vivo imaging system at 6, 12, 24, 36, 48, 72, 96 and 120 h post-injection. Then mice were sacrificed and the major organs and tumor were excised and imaged at 120 h post-injection. The antitumor efficacy and safety evalution experiments were carried out as the method reported by our group.^[4]^ And the tumor-bearing mice were divided into four groups (n=6): normal saline (blank control), (2) Taxol, (3) IPC NPs, and (4) IPS NPs groups. The injection dose and frequency were designed as 15 mg kg^-1^ and 5 times (every other day).

**3. Supporting figures**

**
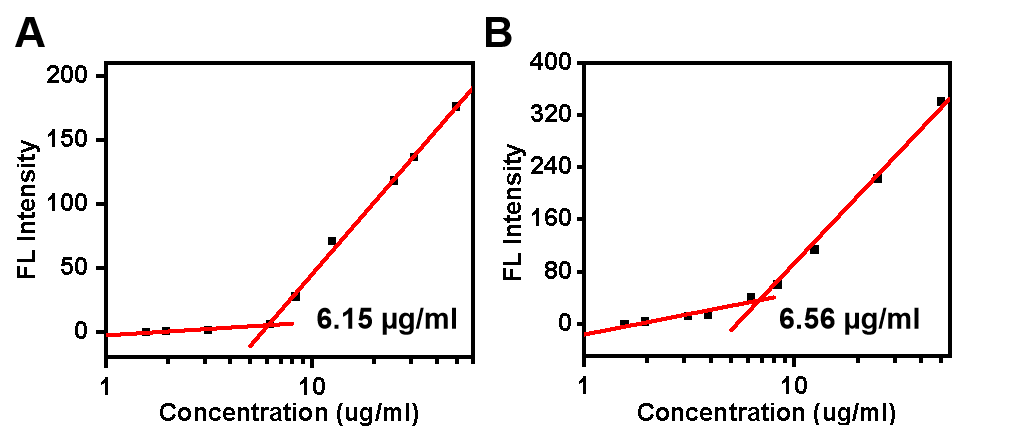
**

**Figure S1.** The critical aggregate concentration of IPS NPs (A) and IPC NPs (B).


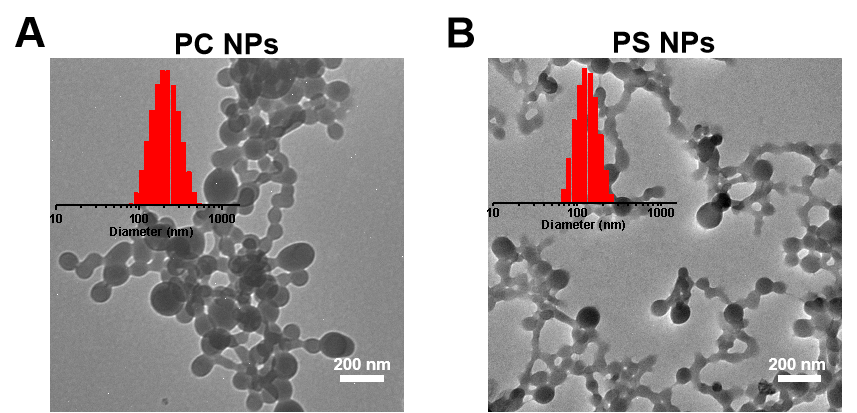


**Figure S2.** The TEM images and size distribution (top left inset) of PC NPs (A) and PS NPs (B).


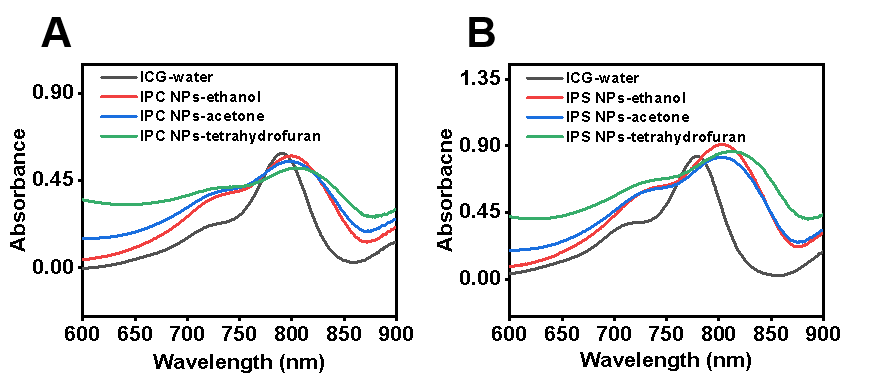


**Figure S3.** The absorption spectra of IPC NPs (A) and IPS NPs (B) obtained by using different organic solvent (ethanol, acetone and tetrahydrofuran).

**Figure S4.** The size distribution of IPS NPs after storage at room temperature for 2 months.


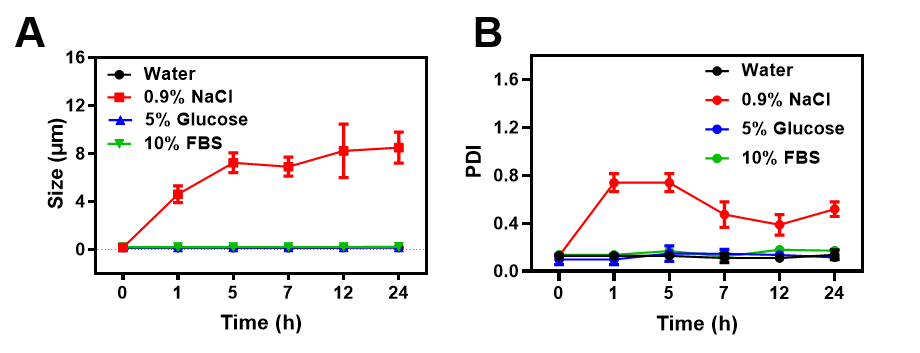


**Figure S5.** (A) Size and (B) PDI changes of PS NPs after incubation with water, 0.9% NaCl, 5% glucose and PBS (pH 7.4) containing 10% FBS for 24 h.


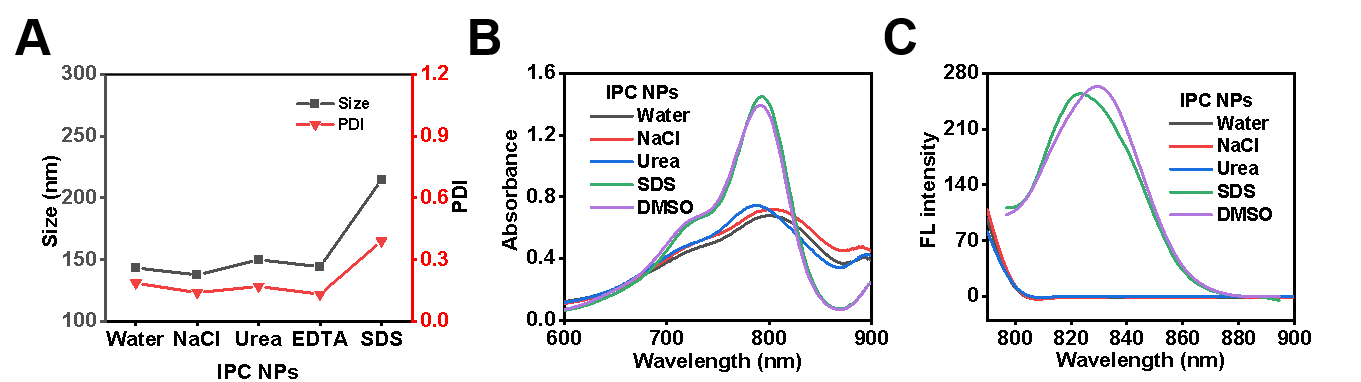


**Figure S6.** (A) Changes of size and PDI of IPC NPs treated with water, NaCl, urea, EDTA and SDS for 24 h. Changes of absorption (B) and fluorescence emission (C) spectra of IPC NPs in different treating solutions (water, NaCl, urea, SDS and DMSO).


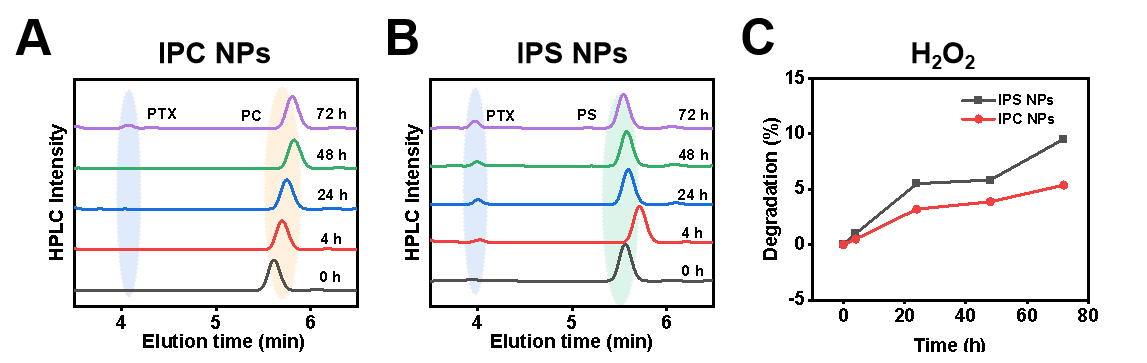


**Figure S7.** Oxidation responsiveness of IPC NPs (A) and IPS NPs (B) upon incubation with 10 mM H_2_O_2_ as determined by HPLC. (C) The degradation rates of IPS NPs and IPC NPs upon incubation with 10 mM H_2_O_2_.


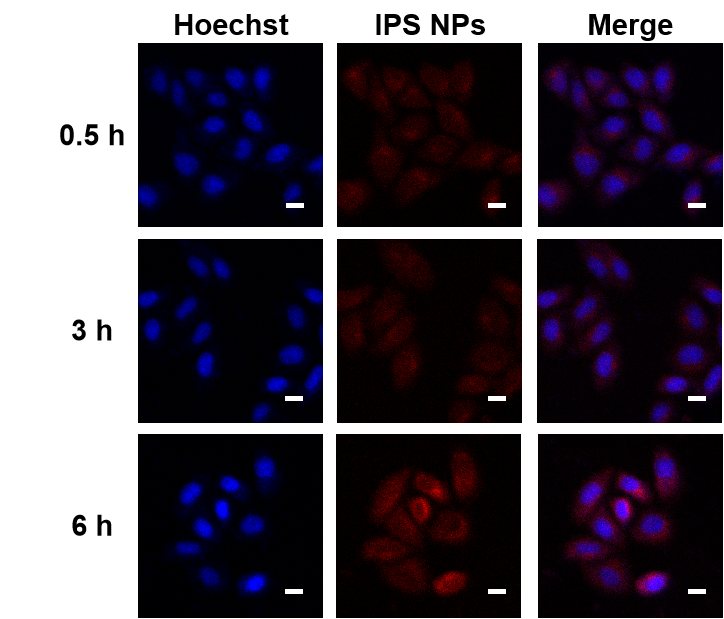


**Figure S8.** CLSM images of HeLa cells incubated with IPS NPs for different times. Scale bars, 20 μm.


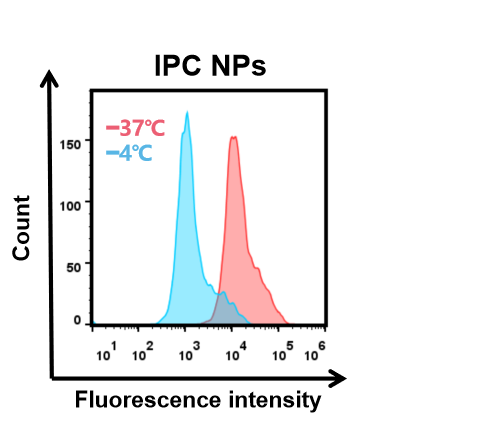


**Figure S9.** FCM fluorescence quantification of endocytosis of IPC NPs at 4 ℃ and 37 ℃.


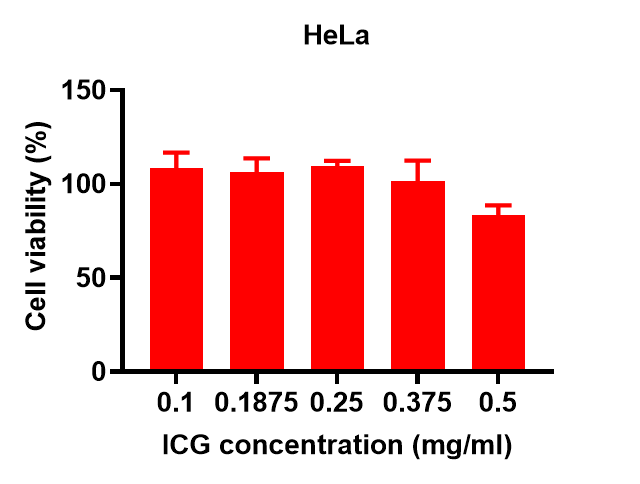


**Figure S10.** *In vitro* cytotoxicity of ICG against HeLa cells for 24h.


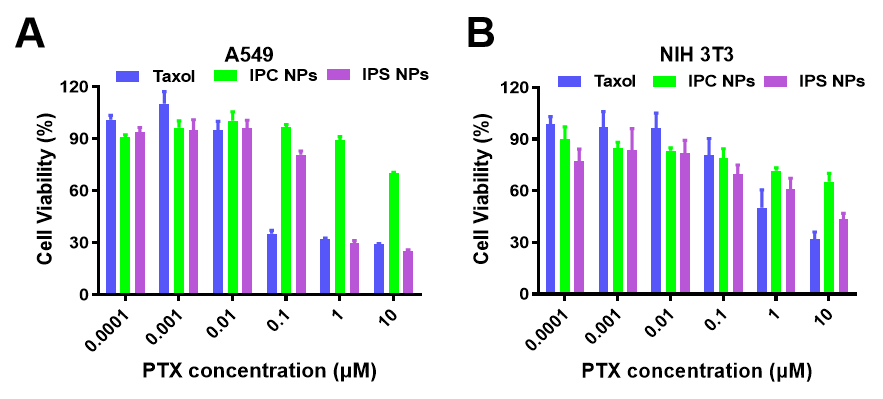


**Figure S11.** *In vitro* cytotoxicity of Taxol, IPC NPs and IPS NPs against A549 cells (A), and NIH 3T3 (B) for 48 h.


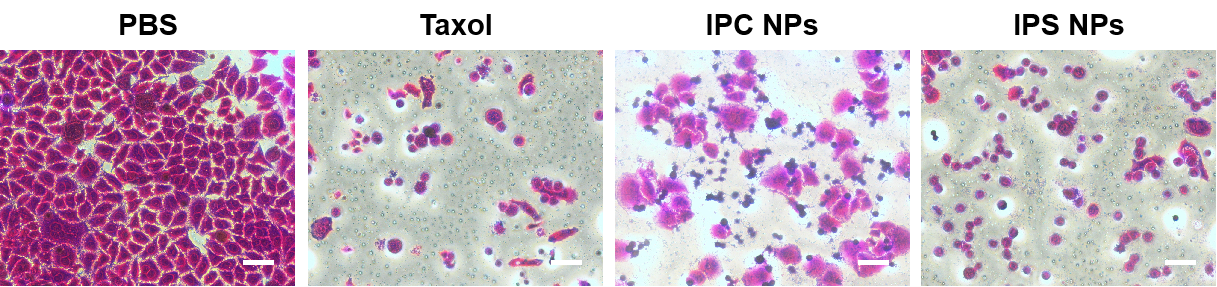


**Figure S12.** Crystal violet staining pictures of HeLa cells incubated with PBS, Taxol, IPC NPs and IPS NPs at equivalent PTX concentration of 10 μM for 48 h. Scale bars, 100 μm.


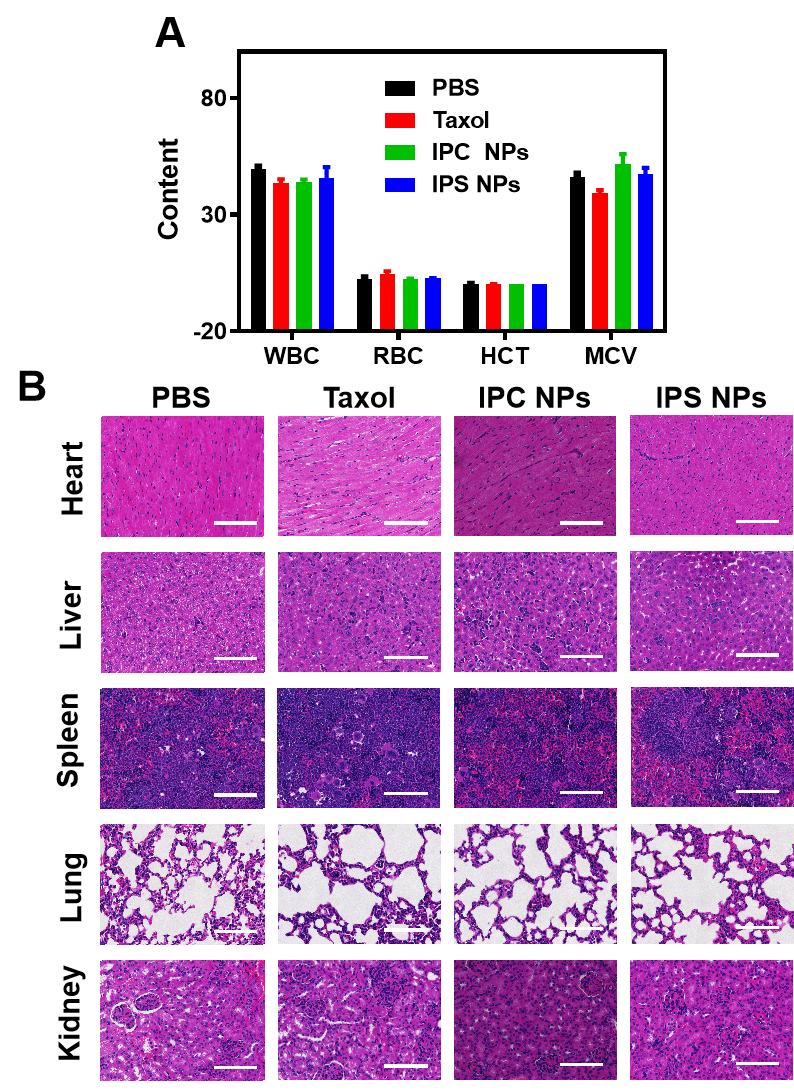


**Figure S13.** (A) Routine blood analysis of mice in PBS, Taxol, IPC NPs, and IPS NPs group. Bars expressed SD (n=3). (B) Histological H&E staining images of major organs of the treated mice. Scale bars, 100 μm.


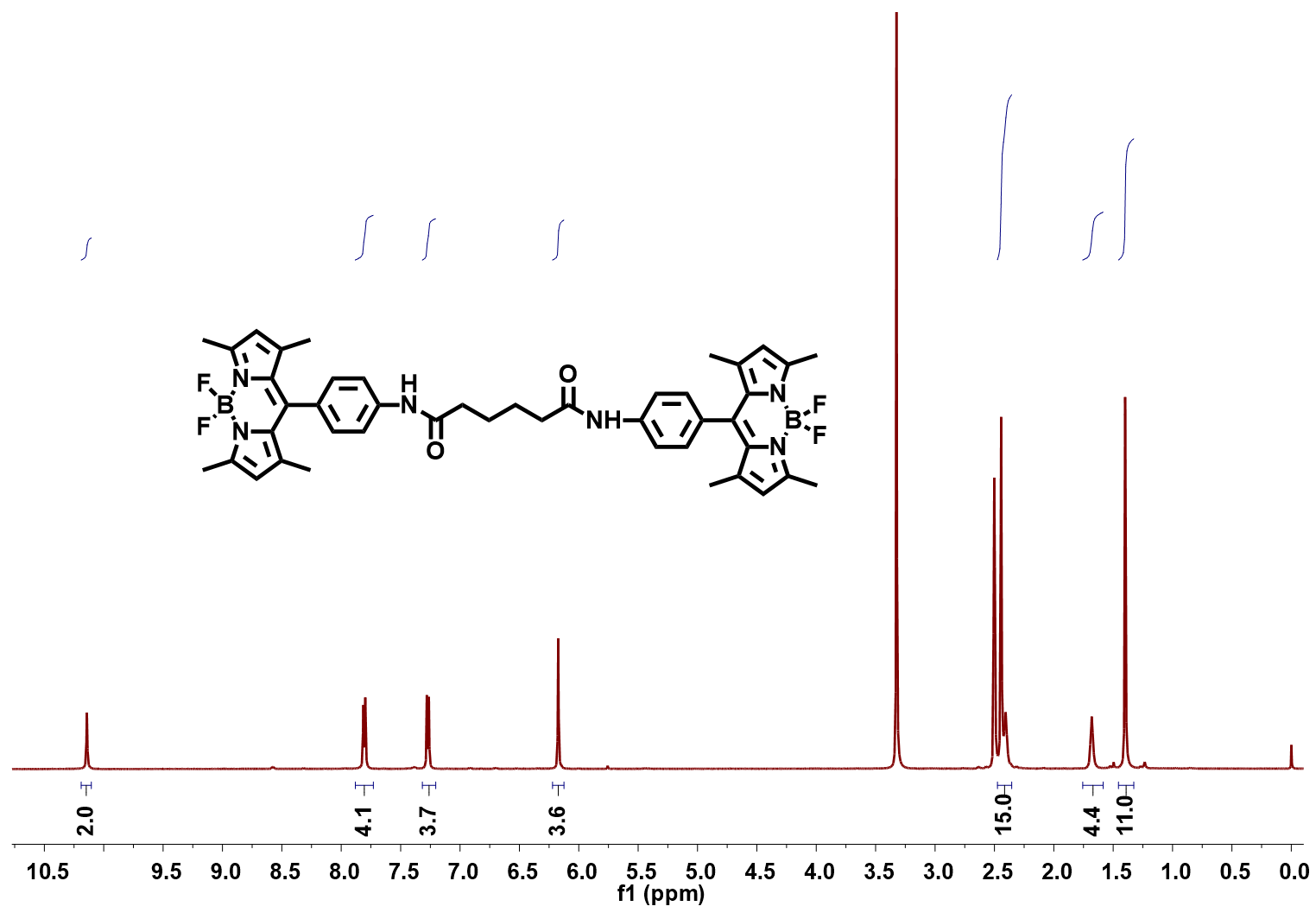


**Figure S14.** ^1^H NMR spectrum of BDP_2_-C6 in DMSO-d6.


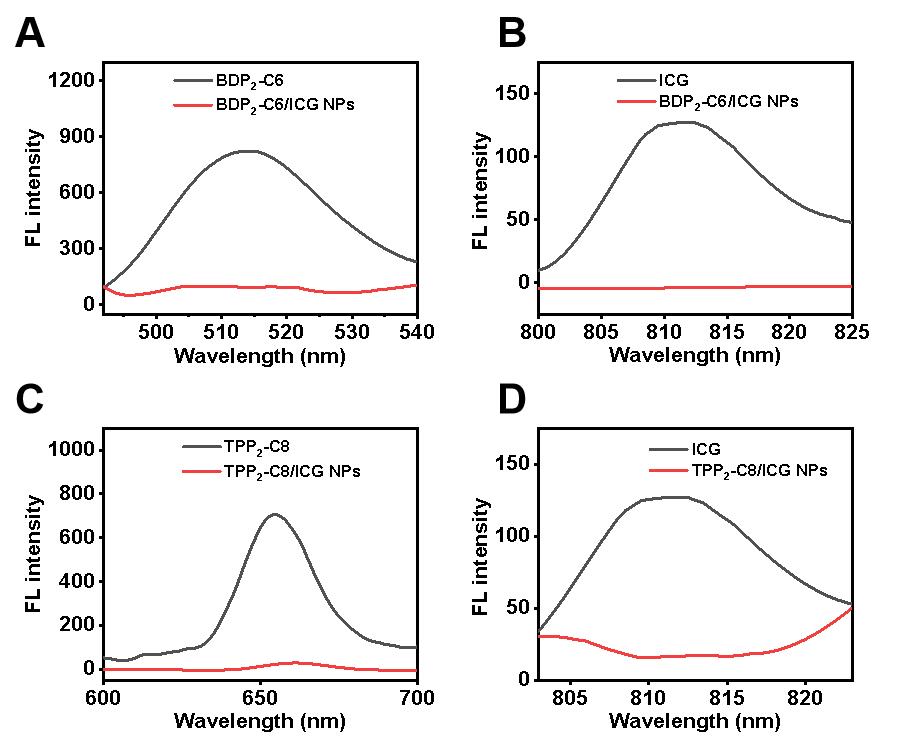


**Figure S15.** (A) Fluorescence emission spectra of BDP fluorophore in BDP_2_-C6 (DMF), and BDP_2_-C6/ICG NPs (water). (B) Fluorescence emission spectra of ICG fluorophore in ICG, BDP_2_-C6/ICG NPs aqueous solutions. (C) Fluorescence emission spectra of porphyrin fluorophore in TPP_2_-C8 (DMF), TPP_2_-C8/ICG NPs (water). (D) Fluorescence emission spectra of ICG fluorophore in ICG, TPP_2_-C8/ICG NPs aqueous solutions.

**References**

[1] J. Wang, Q. Pei, R. Xia, S. Liu, X. Hu, Z. Xie, X. Jing, *Chem. Mater.* **2020**, *32*, 10719;

[2] W. Zhang, W. Lin, X. Zheng, S. He, Z. Xie, *Chem. Mater.* **2017**, *29*, 1856.

[3] A. Vázquez-Romero, N. Kielland, M. J. Arévalo, S. Preciado, R. J. Mellanby, Y. Feng, R. Lavilla, M. Vendrell, *J. Am. Chem. Soc.* **2013**, *135*, 16018.

[4] Q. Pei, S. Lu, J. Zhou, B. Jiang, C. Li, Z. Xie, X. Jing, *ACS Appl. Mater. Interfaces* **2021**, *13*, 59708.
